# Supplementary material for: Characterization of Beef Coming from Different European Countries through Stable Isotope (H, C, N, and S) Ratio Analysis
Source: Molecules. 2023 Mar 22;28(6):2856. doi: 10.3390/molecules28062856 (PMC10057950; doi:10.3390/molecules28062856)
Supplement: Supplementary file 1 [file molecules-28-02856-s001.zip › Table S1.pdf]

Table S1: Inter-laboratory reproducibility for the beef muscle powder (NIST 8414) and the beef liver powder (NIST 1577b) Inter-Comparison Materials (SR = standard deviation of reproducibility).

	**LGL**	**FEM**	**EFS**	**CSL**	**IFR**	**ISO**	**ARC**	**Mean**	**SR**
**NIST 8414**									
$\delta(^{13}C)$ (‰, vs V-PDB)	-25.2	-25.4	-25.5	-25.3	-25.2	-25.3	-25	-25.3	0.2
$\delta(^{15}N)$ (‰, vs AIR)	6.8	6.8	6.6	6.7	6.5	6.4	6.8	6.7	0.2
$\delta(^{2}H)$ (‰, vs V-SMOW)	-147	-146			-149	-143		-146	2.7
$\delta(^{34}S)$ (‰, vs CDT)	5.1	5.4			5.3	5.3		5.3	0.1
**NIST 1577b**									
$\delta(^{13}C)$ (‰, vs V-PDB)	-21.5	-21.6	-21.8	-21.4	-21.4	-21.5		-21.5	0.2
$\delta(^{15}N)$ (‰, vs AIR)	7.6	7.8	7.4	7.5	7.5	7.3		7.5	0.2
$\delta(^{2}H)$ (‰, vs V-SMOW)	-134	-131			-137	-130		-133	3.1
$\delta(^{34}S)$ (‰, vs CDT)	7.5	8.0				7.6	7.1	7.6	0.4

LGL - Bayerisches Landesamt für Gesundheit und Lebensmittelsicherheit, 85764 Oberschleissheim, Germany
FEM - Fondazione Edmund Mach, 38096 San Michele all'Adige, Trento, Italy
EFS - Eurofins Scientific Analytics, BP 42301, 44323 Nantes Cedex 3, France
CSL - The Food and Environment Research Agency, York YO41 1LZ, United Kingdom
IFR - Institute of Food Research, Colney, Norwich N R4 7 UA, United Kingdom
ISO - Isolab Isolab GmbH, 85301 Schweitenkirchen, Germany
ARC - AIT Austrian Institute of Technology GmbH, 2444 Seibersdorf, Austria
