# Supplementary material for: Characterization of Beef Coming from Different European Countries through Stable Isotope (H, C, N, and S) Ratio Analysis
Source: Molecules. 2023 Mar 22;28(6):2856. doi: 10.3390/molecules28062856 (PMC10057950; doi:10.3390/molecules28062856)
Supplement: Supplementary file 1 [file molecules-28-02856-s001.zip › Table S2.pdf]

Table S2: Mean measured $\delta(^2H)$ for each sampling point and estimated $\delta(^2H)$ of water at beef sampling location according to the WaterIsotope database (http://wateriso.utah.edu). Coordinates were provided for all sampling points. In case of lack of information regarding the altitude of the sampling points, the coordinates were used as a reference on a topographic map.

	Measured $\delta(^2H)$ ‰ vs V-SMOW	WaterIsotope database $\delta(^2H)$ ‰ vs V-SMOW	Latitude (DD)	Longitude (DD)	Altitude (m, a.s.l.)
Allgäu (Germany, DE)	-116.3	-75.0	47.6	10.3	1000
Mühlviertel (Austria, A)	-110.3	-63.0	48.0	14.0	450
Frankonia (Germany, DE)	-108.8	-63.0	49.9	11.1	500
Gäuboden (Germany, DE)	-107.1	-66.0	49.0	12.6	500
Bohernagore (Republic of Ireland, IE)	-102.1	-43.0	52.4	-8.3	200
Trento (Italy, IT)	-101.5	-53.0	46.1	11.1	200
Chalkidiki (Greece, GR)	-99.0	-44.0	40.0	23.9	100
Limousine (France, FR)	-98.9	-52.0	45.9	2.0	600
Barcelona (Spain, ES)	-97.0	-33.0	41.4	2.2	12
Orkneys (United Kingdom)	-96.9	-54.0	59.0	-3.1	20
Florence (Italy, IT)	-91.1	-45.0	43.9	11.4	450
Cornwall (United Kingdom)	-90.0	-40.0	50.1	-5.6	110
Sicily (Italy, IT)	-87.0	-44.0	37.8	14.6	800
